# Megahertz-wave-transmitting conducting polymer electrode for device-to-device integration

**DOI:** 10.1038/s41467-019-08552-z

**Published:** 2019-02-08

**Authors:** Taehoon Kim, Gwangmook Kim, Hyeohn Kim, Hong-Jib Yoon, Taeseong Kim, Yohan Jun, Tae-Hyun Shin, Shinill Kang, Jinwoo Cheon, Dosik Hwang, Byung-wook Min, Wooyoung Shim

**Affiliations:** 1 Department of Materials Science and Engineering, Yonsei University, Seoul, 03722 Korea; 2 Center for Multi-Dimensional Materials, Yonsei University, Seoul, 03722 Korea; 3 School of Electrical and Electronic Engineering, Yonsei University, Seoul, 03722 Korea; 4 Yonsei-IBS Institute, Yonsei University, Seoul, 03722 Korea; 5 Center for NanoMedicine, Institute for Basic Science (IBS), Seoul, 03722 Korea; 6 School of Mechanical Engineering, Yonsei University, Seoul, 03722 Korea; 7 Department of Chemistry, Yonsei University, Seoul, 03722 Korea

## Abstract

The ideal combination of high optical transparency and high electrical conductivity, especially at very low frequencies of less than the gigahertz (GHz) order, such as the radiofrequencies at which electronic devices operate (tens of kHz to hundreds of GHz), is fundamental incompatibility, which creates a barrier to the realization of enhanced user interfaces and ‘device-to-device integration.’ Herein, we present a design strategy for preparing a megahertz (MHz)-transparent conductor, based on a plasma frequency controlled by the electrical conductivity, with the ultimate goal of device-to-device integration through electromagnetic wave transmittance. This approach is verified experimentally using a conducting polymer, poly(3,4-ethylenedioxythiophene)-poly(styrenesulfonate) (PEDOT:PSS), the microstructure of which is manipulated by employing a solution process. The use of a transparent conducting polymer as an electrode enables the fabrication of a fully functional touch-controlled display device and magnetic resonance imaging (MRI)-compatible biomedical monitoring device, which would open up a new paradigm for transparent conductors.

## Abstract (web summary)

The emergence of applications requiring device-to-device interactivity has to the need to develop conducting electrodes with high optical transparency at low radiofrequencies. Here, the authors demonstrate conductive polymer electrodes with high transparency in the MHz-order frequency range.

## Introduction

By combining the Ampere–Maxwell equation with Ohm’s law, it would appear that electrical conductivity and optical transparency are incompatible, as determined from the damped wave equation given as *E* = *E*_0_ *e*^−*i*(*ωt*−*z*/*δ*)^ *e*^*−*^^*z*/*δ*^, where *E* is the electric field of the traveling wave, *E*_0_ is the electric field amplitude at the surface, *ω* is the angular frequency, *t* is the time, *z* is the distance traveled, and *δ* is the skin depth^1^. When visible light enters a metallic conductor as an electromagnetic wave, most of the incident energy is reflected, with penetration being in the order of a few atoms only, as defined by the skin depth *δ* = (2/*ωμσ*)^1/2^, where *μ* is the permeability of the conductor and *σ* is the electrical conductivity. Therefore, metals are opaque to visible light (429 THz < *ω*_visible light_/2π < 750 THz).

**Fig. 1:**
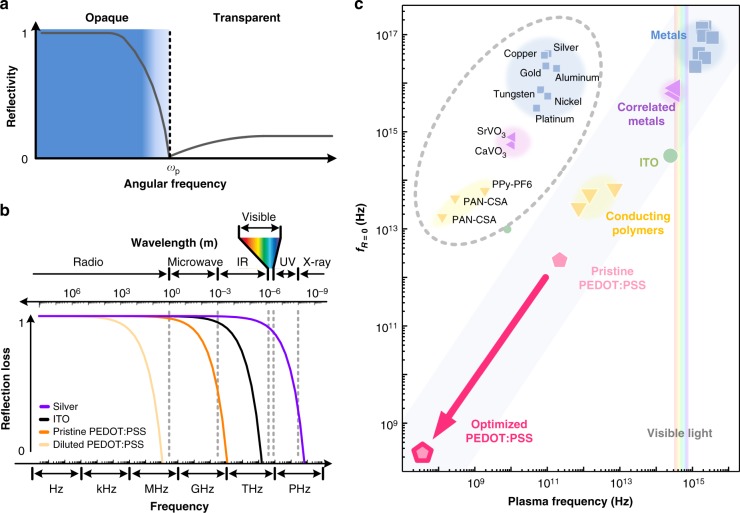
Relationship between plasma frequency and reflection for transparent conductors. **a** Schematic of increase in light transmission (decrease in reflectivity) of metals for frequencies beyond the plasma frequency. **b** Frequency-dependent reflection loss *R* of four materials (silver, indium tin oxide (ITO), pristine poly(3,4-ethylenedioxythiophene):-poly(styrenesulfonate) (PEDOT:PSS), and diluted PEDOT:PSS) calculated by intrinsic impedance. **c** Correlation between vertical intercept (*R* = 0) of reflection loss curve, *f*_*R*=0_, calculated by *σ* and plasma frequency *ω*_p_ that is proportional to electron concentration *n*

The minimum frequency at which an electromagnetic wave can pass through a conductor, represented by the plasma frequency *ω*_p_ = (*ne*^2^/m^*^ε)^1/2^, where *e* is the electron charge, m^*^ is the effective electron mass, and *ε* is the permittivity of the conductor, can be tuned by controlling the free electron concentration *n*. For a Drude conductor, the electrical conductivity *σ* = *enμ*_*e*_ = *e*^2^*τ*(*n*/m^*^), with electron mobility *μ*_*e*_ and scattering time *τ*, also having to be controlled, making the ratio *n*/m^*^ the key factor affecting the optimization of the transparency of the conductors to electromagnetic waves^2^. Indeed, electromagnetic waves with frequencies greater (smaller) than *ω*_p_ will pass through (be blocked by) the conductors (Fig. 1a), i.e., the conductor becomes transparent or opaque at sufficiently high and low frequencies, respectively. In general, the former has often been cited in proposed design strategies for identifying a transparent conducting electrode^3,4^, while the latter can be used to identify materials that provide shielding against electromagnetic interference (EMI)^5–7^. As the scale of electronics and the constituent components becomes smaller, while operating speeds increase, a substantial increase in EMI has arisen^8^, leading to issues with malfunctions and degraded levels of performance^9–13^. This increase in electromagnetic wave leakage can also pose a problem to surrounding electronics if no shielding is provided^14^.

Contrary to the conventional wisdom of EMI shielding materials^5–8^, by focusing on signaling rather than leakage of electromagnetic waves, the use of no EMI shielding^15,16^ is emerging as an important field of research related to ‘device-to-device interactivity’. With increasing demand for complex, multifunctional electronics having a compact format, the ability to convert one type of signal (electricity^17–19^, force^20–23^, light^24–27^) into a different form is an attractive feature. This can be realized by integrating separate single-function devices into one, and, as such, it is crucial to maintain each device’s functionality and the ability to perform two processes once integrated. Otherwise, the design would require twice as many devices and a much more cumbersome fabrication process. To this end, when two functional devices are integrated, one device must be transparent to certain electromagnetic (EM) waves (i.e., radiofrequencies of 20 kHz–300 GHz) while also containing conducting electrodes that fundamentally shield against the EM waves emitted by the other device. In general, metal is found to be transparent to EM waves with a frequency greater than 1 PHz (that is, *ω*_p,metal_ ≈ 100 THz)^28^, and thus is not transparent to radiofrequencies (1 GHz–1 THz). This has prompted researchers to consider the possibility of designing a conducting material for which *ω*_p_ is well below the radiofrequency range. This, however, has proven to be challenging to realize; it requires the design of a material that can both conduct electricity and transmit EM waves at low frequencies (much lower than visible light).

Herein, we report on a concept aimed at realizing ‘device-to-device integration’ through the use of polymer conductors that are made transparent to low-frequency waves by lowering the plasma frequency *ω*_p_ modulated by the polymer microstructure. Compared to a transparent conducting oxide (TCO), polymer conductors that are transparent to low-frequency waves offer many potential advantages in terms of transparency to a wide frequency range, scalability, and potential applications. First, poly(3,4-ethylenedioxythiophene):poly(styrenesulfonate) (PEDOT:PSS), which is transparent to MHz-order frequencies, is proposed by shifting the plasma frequency below the microwave range (<1 GHz). Second, because the process involves the application of solution-based polymer chemistry, there is the potential for aggressive device scaling without costly processing steps, unlike the case with a TCO. Third, an EM-transmittable pressure-sensor interface, integrated into a touch-control display and medical image scanner, demonstrates a multifunctional device-to-device architecture. Although the pressure-sensor application provides a starting point for our research, we also focus on the analysis of how this material can lead to designs for ‘device-to-device integration.’ We first describe the design of the conductor material, which is transparent to low frequencies, then present its optical and electrical performance, in order to demonstrate that the integration of this electronic element into a large-scale prototype display interface is feasible for application as a three-dimensional (3D) touch-interactive system and magnetic resonance imaging (MRI)-compatible pressure sensor for medical imaging.

## Results

### Approach to low-frequency transparent conductor

We first determined the reflection loss of various materials (e.g., silver, indium tin oxide (ITO), and PEDOT:PSS of different concentrations) based on the frequency-dependent intrinsic impedance^29^. When an electromagnetic wave passes from air (*η*_0_) to another medium (*η*), the intensity of the reflected part of the incident wave is represented by a reflection coefficient: *Γ*_air/medium_ = (*η*_0_ − *η*)/(*η*_0_ + *η*), where *η*_0_ is the intrinsic impedance of the air (377 Ω) and *η* is the intrinsic impedance of the medium. If an electromagnetic wave passes through a free-standing film, the intensity of the transmitted wave decreases at a ratio of *T* = |(1 − *Γ*_air/film_)(1 − *Γ*_film/air_)| across the two interfaces. Therefore, the loss ratio due to the reflection *R* is calculated from *R* = 1 − *T* = 1 − |(1 − *Γ*_air/film_)(1 − *Γ*_film/air_)|. When the electromagnetic wave completely passes through the material (i.e., the material is transparent), the reflection loss is zero (*R* = 0). The vertical intercept, *f*_*R* = 0_, provides an estimate of *ω*_p_ that is proportional to the electron concentration *n*, as shown in Fig. 1b. The intercept decreases with the value of *n*, corresponding to a decrease in *σ*. As *ω*_p_ for metal for which *n* = 10^22^/cm^3^ and *σ* = 10^7^ S/m is above the visible range (purple curve, Fig. 1b), semiconductors with a lower *n* and *σ* can be considered for the development of a TCO (black curve, Fig. 1b). ITO is widely used for transparent electrodes, which indeed have a plasma frequency in the near-infrared range (black curve), allowing it to transmit visible light while still having a usable level of conductivity (ca. 10^5^ S/m; 1% of the conductivity of the metal). To attain transparency at lower frequencies, conducting polymers can be considered. For example, pristine PEDOT:PSS for which *σ* *=* 10^3^ S/m has a plasma frequency in the far-infrared range (<20 THz, orange curve, Fig. 1b), which allows the transmission of visible light while maintaining conductivity. As a means of attaining ‘device-to-device interactivity,’ we propose a PEDOT:PSS that is transparent to MHz frequencies, making it more transparent by shifting the plasma frequency to below the microwave range (<1 GHz, yellow curve, Fig. 1b), corresponding to *σ* *=* 10^−1^ S/m; rather than changing the film thickness, the electrical conductivity *σ* is utilized to optimize *ω*_p_ (Supplementary Fig. 1). Figure 1c shows the room-temperature conductivities and plasma frequencies reported for metals^30–32^ (Ag, Cu, Au, Al, W, Ni, and Pt), correlated metals^33^ (SrVO_3_ and CaVO_3_), and conducting polymers^34^ (PPy-PF_6_, PAN-CSA, and pristine PEDOT:PSS). This simple correlation between *σ* and *ω*_p_ enables extrapolation to determine those conducting polymers that are transparent to MHz frequencies (Fig. 1c, lower-left corner); the small *σ* makes it likely that *ω*_p_ will decrease, such that the correlation between *σ* and *ω*_p_ is obvious. Indeed, based on our calculations (Supplementary Note 1), a PEDOT:PSS that is transparent to MHz frequencies should have a *σ* value of 10^−1^ S/m, which is still higher than that of pure silicon (<10^−2^ S/m). As such, this approach moves the state of the art closer to the development of conductors that are transparent to low-frequency electromagnetic waves.

**Fig. 2:**
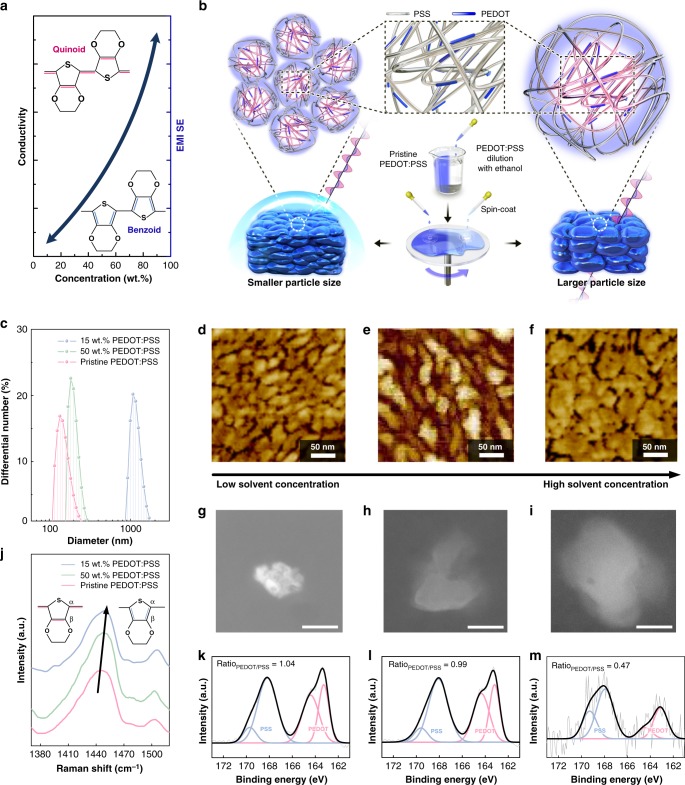
Process and characteristics of MHz frequency-transparent poly(3,4-ethylenedioxythiophene):-poly(styrenesulfonate) (PEDOT:PSS) film. **a** Interrelationship between conductivity, electromagnetic interference (EMI) shielding efficiency (SE), and PEDOT:PSS concentration of the solution. **b** Schematic of fabrication of the PEDOT:PSS films with different microstructure and electromagnetic (EM) characteristics. As the solvent concentration increased, larger particle size and higher EM wave transmittance is obtained. **c** Particle size distribution of 15 wt.%, 50 wt.% PEDOT:PSS/ethanol solutions and pristine PEDOT:PSS aqueous solution. **d**–**f** Atomic force microscopy (AFM) phase images of spin-coated film produced using **d** pristine PEDOT:PSS, **e** 50 wt.%, and **f** 15 wt.% PEDOT:PSS/ethanol solution. **g**–**i** High-angle annular dark-field scanning transmission electron microscopy (HAADF-STEM) images of drop-casted film produced using **g** pristine PEDOT:PSS, **h** 50 wt.%, and **i** 15 wt.% PEDOT:PSS/ethanol solution. The scale bar corresponds to 50 nm. **j** Raman spectroscopy of PEDOT:PSS film spin-coated with different PEDOT:PSS concentrations. The peak attributed symmetrical stretching vibration is shifted from 1443 cm^−1^ to 1448 cm^−1^, indicating the transformation of the PEDOT from a quinoid structure to a benzoid structure. **k**–**m** X-ray photoelectron spectroscopy (XPS) spectra of sulfur 2p for spin-coated film using **k** pristine PEDOT:PSS, **l** 50 wt.%, and **m** 15 wt.% PEDOT:PSS/ethanol solution. The PEDOT:PSS intensity ratio decreases as the concentration decreases

We next rationalized that transparency to MHz frequencies requires a large PEDOT:PSS particle size, as typically seen in materials with a low conductivity and low EMI characteristics (Fig. 2a). In general, as the PEDOT:PSS particle size become smaller (larger), it leads to an increase (decrease) in the contact area between the particles, resulting in an enhancement (reduction) in the conductivity^35^. In this regard, we controlled the particle size by adjusting the solvent concentration; the more diluted PEDOT:PSS, the larger particle size (Fig. 2b). Qualitatively, there are several factors contributing to the conductivity of PEDOT:PSS. First, we investigated how the solvent affects the particle size, given that the solubility of a polymer depends on the solubility parameter (*δ*) defined by the Hildebrand–Scatchard equation. For a binary system, the Hildebrand–Scatchard equation relates the solubility parameters of solvents to the enthalpy change upon their mixing: Δ*H*_m_ = *V*_m_(*δ*_1_ − *δ*_2_)^2^*φ*_1_*φ*_2_, where *V*_m_ is the volume of the mixture, *δ*_*i*_ is the solubility parameter of component *i*, and *φ*_*i*_ is the volume fraction of *i* in the mixture^36^. If swelling is to occur in a PEDOT:PSS-ethanol binary system, the free energy of mixing must be favorable, that is, Δ*G*_m_ < 0. Because Δ*G*_m_ = Δ*H*_m_ − *T*Δ*S*_m_, swelling is a maximum when (*δ*_ethanol_ − *δ*_PEDOT:PSS_)^2^ = 0, where the solubility parameter of PEDOT:PSS is equal to that of ethanol. In this case, we considered the solubility parameters of PEDOT *δ*_PEDOT_ (25 J^1/2^ cm^−3/2^) and PSS *δ*_PSS_ (21.3 J^1/2^cm^−3/2^)^37^ individually so that the degrees of swelling could be estimated separately. The value of *δ*_ethanol_ was found to be 26.2 J^1/2^cm^−3/2^, thereby justifying the selection of ethanol as a good solvent for attaining swelling. Indeed, the particle size distribution of pristine PEDOT:PSS in water (*δ*_water_ = 47.9 J^1/2^cm^−3/2^)^36^ and diluted PEDOT:PSS is clearly dependent on the solvent selection and its mixture ratio. Figure 2c shows that the particle sizes gradually increased to 136 ± 40 nm (red curve), 185 ± 45 nm (green curve), and 1076 ± 220 nm (blue) when using water (pristine PEDOT:PSS solution), 50 wt.%, and 85 wt.% ethanol as solvents, respectively, as measured using a particle size analyzer of the colloidal solutions. Importantly, the particle size after spin-coating and drying becomes smaller and flat, which allows for uniform stacking, and as such can be used as a film for conducting electrodes. We unambiguously characterized the surface morphology of the spin-coated film using atomic force microscopy (AFM); the phase images of the PEDOT:PSS films show that the particle size increased to 35 ± 10 nm (Fig. 2d), 45 ± 10 nm (Fig. 2e), and 65 ± 15 nm (Fig. 2f) when using water, 50 wt.%, and 85 wt.% ethanol as solvents, respectively. Note that the PEDOT:PSS particles have a flattened pancake-like morphology rather than a spherical shape after annealing^38^, whereby stacking of the particles forms a film. This trend is also in good agreement with the images obtained from high-angle annular dark-field (HAADF) scanning transmission electron microscopy (STEM), as shown in the Fig. 2g–i.

Second, we investigated how increasing the amount of ethanol solvent affects the structure of the PEDOT:PSS in the quinoid (bipolaron state) and benzoid (neutral state) forms. The quinoid of PEDOT typically exhibits a delocalized state of π-electrons and, thus, there will be a high *σ* value owing to the formation of carbon–carbon double bonds between adjacent thiophene rings, while π-electrons are localized for the benzoid (single-bond), leading to a low value of *σ*^39^. In organic molecules, the first carbon attached to the functional group is referred to as *C*_α_, and the second carbon as *C*_β_. The Raman spectra, shown in Fig. 2j, shows the different vibration modes inside a thiophene ring in PEDOT:PSS when the solvents are changed; there is a slight shift from *C*_α_ − *C*_β_ inside the thiophene ring (quinoid) to *C*_α_ = *C*_β_ inside the thiophene ring (benzoid) (the inset of Fig. 2j), from 1443 cm^−1^ (water) to 1448 cm^−1^ (85 wt.% ethanol). This observation reveals an obvious transformation from a quinoid structure to a benzoid structure, as observed in studies^40^, where the transition of the PEDOT chains from the bipolaron (high *σ*) to neutral state (low *σ*) takes place (Supplementary Fig. 2). This may be related to the pH; as the pH increases, more quinoid is transformed into benzoid, corresponding to a decrease in the amount of bipolaron and polaron^41^. Indeed, the pH values for the pristine PEDOT:PSS aqueous solution, 50 and 15 wt.% PEDOT:PSS/ethanol were 2.34, 2.81, and 3.54 (Supplementary Fig. 3). Third, as noted in the discussion of PEDOT-solvent compatibility, PSS (*δ*_PSS_ = 21.3 J^1/2^cm^−3/2^) is also likely to swell in ethanol (*δ*_ethanol_ = 26.2 J^1/2^cm^−3/2^). Figure 2k–m shows X-ray photoelectron spectroscopy (XPS) data for pristine, 50 wt.%, and 15 wt.% PEDOT:PSS, which indicate that the binding energy ratio of PEDOT:PSS decreased by approximately 50% (relative to the ratio for pristine PEDOT:PSS). Note that the XPS band at lower binding energies of 163–166 eV corresponds to the sulfur atoms in PEDOT, while the higher binding energy near 169 eV corresponds to the sulfur atoms in the PSS dispersant^42^. In fact, the reduction of the PEDOT:PSS intensity ratio of S 2p can be clearly seen: 1.04 (pristine, Fig. 2k), 0.99 (50 wt.%, Fig. 2l), and 0.47 (15 wt.%, Fig. 2m). Given that the photon energy *hν* (Al Kα line: 1486.6 eV) from the XPS gun does not vary when examining the surface of the PEDOT:PSS, the decreased intensity ratio can be attributed to the PSS swelling such that the conductivity falls (Supplementary Fig. 4)^40^, which is also consistent with the resulting low *σ* and, consequently, the high optical transparency.

**Fig. 3:**
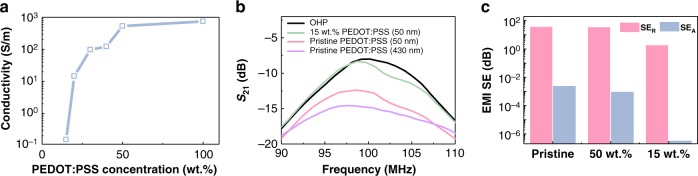
Electrical and electromagnetic (EM) characteristics of poly(3,4-ethylenedioxythiophene):-poly(styrenesulfonate) (PEDOT:PSS) films of different concentrations. **a** Electrical conductivity of electrode as a function of PEDOT:PSS concentration of solution. The inset shows a 4-point probe that measures the sheet resistance of a PEDOT:PSS film. **b** Measured *S*_21_ parameter of OHP/PEDOT:PSS films of different thicknesses and solution concentrations over a 90–110-MHz frequency range. Even if a same thickness of PEDOT:PSS is coated, the EM wave transmittance can vary greatly depending on the concentration of solutions. **c** Calculated SE_R_ and SE_A_, based on the measured conductivities and thicknesses of the PEDOT:PSS films that is spin-coated at different concentrations at 100 MHz

Figure 3a shows a representative 4-point probe measurement (Supplementary Fig. 5) of PEDOT:PSS with different concentrations onto glass slides. The PEDOT:PSS was spin-coated onto the surface, and then baked at 70 °C. The unambiguous demonstration of the ohmic scaling exhibits a constant conductivity *σ* *=* 1/*ρ* *=* 1*/*(*R*_*s*_*∙t*), where *ρ* is the resistivity, *R*_*s*_ is the sheet resistance measured by a 4-point probe, and *t* is the thickness measured via AFM. Following a typical 4-point probe measurement, however, the conductivity of the PEDOT:PSS exhibits thickness-dependent change. For example, the value of *σ* for typical pristine PEDOT:PSS was in a range of 128–744 S/m, similar to those observed in previous studies^40^, whereas the value of *σ* for films of different concentrations decreased from 128 to 0.153 S/m (15 wt.%) for a fixed thickness (50 nm) (Supplementary Fig. 6). Such large changes (a factor of 10^3^) ensure that the microstructures are coupled with the electrical conductivity^35^, and thus maximize the capability to control the structure/property complexity of PEDOT:PSS.

To explore the EM-transmitting properties of PEDOT:PSS, we compared five PEDOT:PSS samples with conductivities of 0.153–744 S/m and a thicknesses of 50–430 nm (Supplementary Fig. 7a). Note that the thickness is much less than the skin depth *δ* for PEDOT:PSS (>hundreds of μm at 100 MHz), and thus absorption is negligible compared to reflection (regardless of the thickness). The transmitting efficiency, *S*_21_, was measured using a vector network analyzer with an overhead projector (OHP)/PEDOT:PSS film between antennas 1 and 2 (Supplementary Fig. 8). Given that EM transmitting is inversely proportional to *σ*, the 15 wt.% PEDOT:PSS with the lowest value of *σ* exhibits the highest degree of EM transmission. With a decrease in the PEDOT:PSS content, *S*_21_ increased (Supplementary Fig. 7b). Importantly, as shown in Fig. 3b, 50 nm layers of pristine and 15 wt.% PEDOT:PSS exhibit substantially different *S*_21_ values (−12.7 and −8.6 dB at 100 MHz, respectively), verifying that the particle size and conductivity are crucial factors affecting EM transmission and that there is no thickness dependence below a skin depth of *δ* (Supplementary Fig. 9). In addition, the EMI shielding efficiency (SE) calculated from the absorption (SE_A_) and reflection (SE_R_) values for the pristine, 50 wt.%, and 15 wt.% PEDOT:PSS were plotted in Fig. 3c at 100 MHz, showing that transmission owing to the suppression of the SE_R_ value was the dominant mechanism (SE_A_ is smaller than SE_R_ by >5 orders of magnitude (dB)). Considering the frequency range of visible light (THz) and microwaves (GHz), it is interesting to note that a conducting polymer can be transparent even to MHz frequencies and still have enough charged electrons to enable its use as a conducting electrode.

### Characteristics of capacitive pressure sensor

The concept of ‘device-to-device interactivity’ has gained considerable attention as a means of enhancing user interfaces and interactive sensors^22,43,44^. These devices display information by sensing external stimuli and simultaneously converting it into a human-readable readout. In terms of applications, this capability can be directly harnessed to offer a platform for the development of stimuli-responsive (e.g., temperature, light, pressure) devices, such as electronic skin (e-skin) and touch interfaces^45^. We set out to develop an EM-transmitting pressure-sensor interface integrated into a touch-controlled display and medical imaging scanner as a proof-of-concept multifunctional device-to-device architecture. The low *σ* value of thin 15 wt.% PEDOT:PSS enables its use as a conducting electrode in a capacitor, as related to the following parameters: the capacitance (*C* *=* *ε*_*r*_*ε*_0_*A*/*d*, where *ε*_*r*_ is the relative permittivity of the dielectric layer, *ε*_0_ is the vacuum permittivity, *A* is the plate area, and *d* is the plate separation) of the pressure sensor depends only on geometrical factors (namely, *A* and *d*), not on *σ*; the depth within the plate where the electrons accumulate is <1 pm (see Supplementary Note 2); and the power consumption of the sensor does not increase with the resistance of the electrode.

**Fig. 4:**
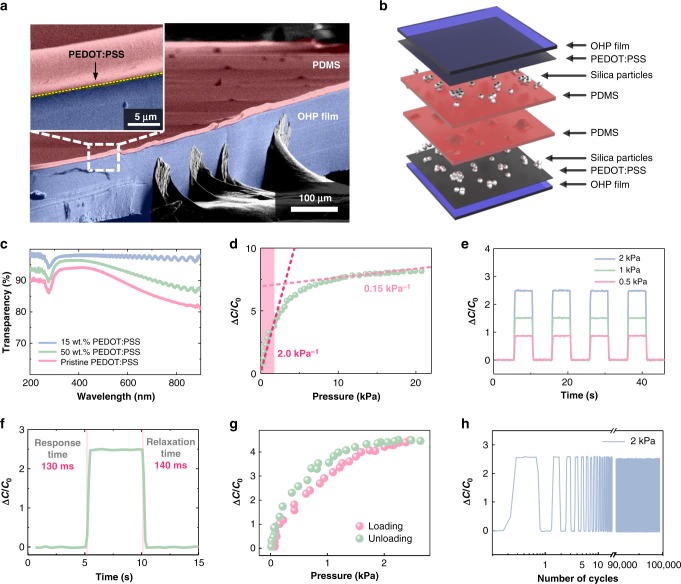
Structure and sensing capabilities of demonstrated capacitive pressure sensor. **a** Tilted-view scanning electron microscopy (SEM) image of sensor layer. **b** Schematic of capacitive pressure sensor consisting of 500-nm diameter silica nanoparticles dispersed in poly(dimethylsiloxane) (PDMS)/poly(3,4-ethylenedioxythiophene):-poly(styrenesulfonate) (PEDOT:PSS) layers on overhead projector (OHP) film substrate. **c** Measured transparency as a function of light wavelength from 200 to 900 nm for different concentrations of PEDOT:PSS/ethanol solution spin-coated onto the sensor layers of OHP substrates. **d** Capacitive response for a pressure of 0–20 kPa applied to the capacitive pressure sensor. **e** Time-resolved capacitive response of a sensor under repeated normal pressure values of 0.5 (red line), 1 (green line), and 2 kPa (blue line). **f** Response and relaxation time after loading (2 kPa for 5 s) and unloading. **g** Relative capacitance change from two consecutive linear loading (2 kPa) and unloading cycles. **h** Stability of capacitive response of the capacitive pressure sensor to a load of 2 kPa over 100,000 cycles

Figure 4a shows a representative false-color scanning electron microscope (SEM) image of a pressure sensor in which PEDOT:PSS is used as a transparent electrode. Briefly, an OHP film (Hansol Paper Co., Ltd.) with a transparency of 97% was used as a transparent and flexible substrate (Fig. 4a, blue). A 50 nm PEDOT:PSS (15 wt.%) layer, applied by spin-coating, acts as the transparent conducting electrode and, subsequently, SiO_2_ nanoparticles (500 nm in diameter) in ethanol were dispersed on top of the PEDOT:PSS layer. A diluted thin poly(dimethylsiloxane) (PDMS) layer, mixed with heptane solution (40 wt.% PDMS in heptane), was applied to the nanoparticle-dispersed surface (Fig. 4a, red) such that the surface roughness formed by nanoparticle aggregation was maintained^46,47^. Thus, a highly transparent single-cell capacitor in which a half-cell consisting of nanoparticles dispersed in a PDMS dielectric layer on the PEDOT:PSS electrode and a PEDOT:PSS counter electrode was subsequently fabricated to yield a metal–insulator–metal sandwich structure (Fig. 4b and Supplementary Fig. 10).

We then investigated the optical and electrical characteristics of the pressure sensors. First, for the 15 wt.% PEDOT:PSS, the optical transmittance exceeded 96.7% in the visible wavelength region (380–760 nm, Supplementary Fig. 11), whereas that of the pristine PEDOT:PSS sensors was <95%, although this is still sufficient to appear transparent (Fig. 4c); such a high level of optical transparency is useful for enabling integration with optical devices. In fact, we previously reported on a design strategy for fabricating high-transparency and high-sensitivity individual capacitive pressure sensors that relied on the multiple states of nanoparticle dispersity^46^. Second, the pressure sensitivity, defined as *S* *=* *δ*(*∆C*/*C*_0_)/*δp*, where *p* is the normal pressure, and *C* and *C*_0_ are the capacitances with and without normal pressure, respectively, was measured (Fig. 4d). The value of *S* of the capacitive pressure sensor was measured at 2.0 kPa^−1^ and 0.15 kPa^−1^ for pressures of <2 kPa and >10 kPa, respectively. The homogeneous dispersion of SiO_2_ nanoparticles leads to the PDMS layer having a rough interface, and thus renders the air gap, which significantly improves the pressure sensitivity, similar to that observed in the microstructured capacitive pressure sensors^46–48^.

We subsequently investigated the pressure-sensing capabilities in terms of the response time. An analysis of the capacitance over time for four cycles, in which a pressure of 0.5, 1, and 2 kPa was applied, revealed that the sensor exhibited a fast response time (Fig. 4e). A reliable and repeatable sensing behavior was observed, whereby the capacitance of the sensor changed sharply with pressure loading and unloading. Specifically, Fig. 4f shows the response and relaxation times of the pressure sensors upon the loading (2 kPa) and unloading of the sensor. The estimated response and recovery times upon loading and unloading, defined as the time constants given by 1 − 1/*e* (approximately 63%) (for response) and 1/*e* (approximately 36%) (for recovery), were 130 and 140 ms, respectively. Given that the SiO_2_ nanoparticles/PDMS form truncated cone-shaped features, the PDMS becomes easily compressible relative to a planar PDMS architecture, which renders it more elastic^46^. Finally, two characteristics, namely, stability and robustness, were characterized for a 1 × 1 cm^2^ pressure sensor. In the former case, the hysteresis, which is the consecutive linear loading/unloading cycle up to 10 kPa in the context of the pressure response curves (Fig. 4g), was small, pointing to the good stability of the sensors. Notably, the sensor endured 100,000 loading/unloading cycles with a load of 2 kPa and maintained its full functionality with no output signal degradation (Fig. 4h).

### Device-to-device integration

**Fig. 5:**
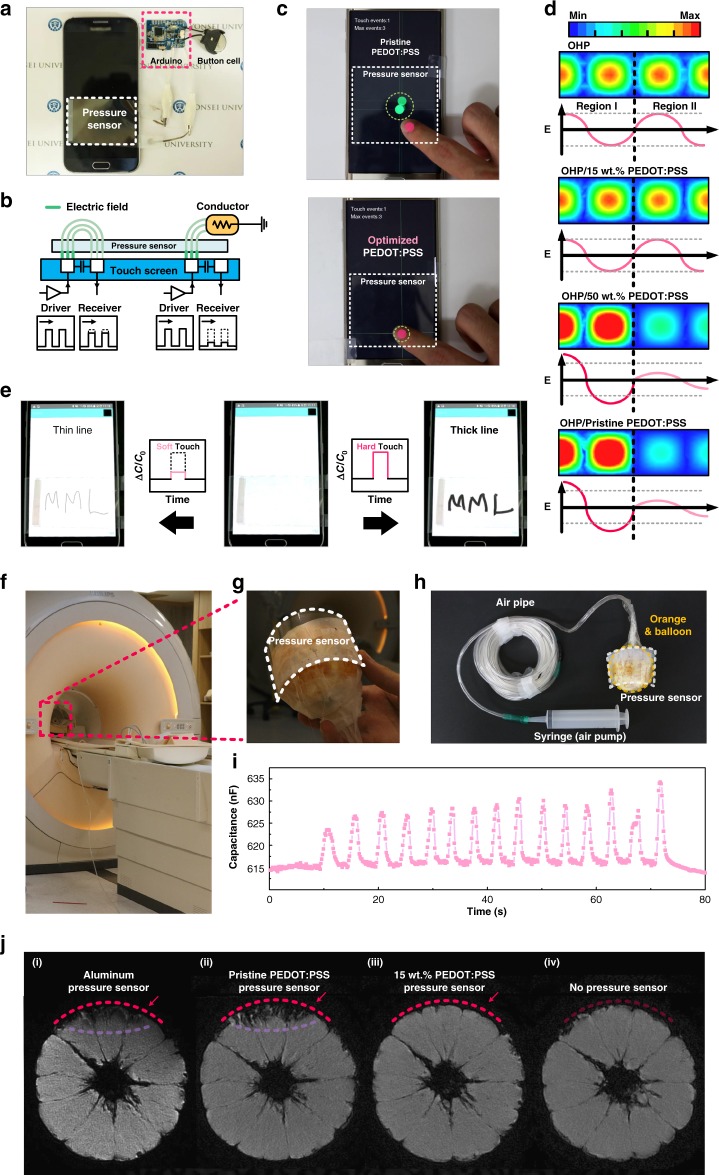
Demonstration of general applicability of pressure sensors. **a** Pressure sensor connected to Arduino board with Bluetooth module. **b** Schematic of electric field distribution when a transparent capacitive pressure sensor using 15 wt.% poly(3,4-ethylenedioxythiophene):-poly(styrenesulfonate) (PEDOT:PSS) is directly attached to a touch device, together with the difference between the ‘untouched’ and ‘touched’ states. **c** Touch accuracy of touchscreen connected to pressure sensors with pristine PEDOT:PSS and 15 wt.% PEDOT:PSS film. The red dot indicates the position of at which the sensor is actually touched, while the green dot indicates the spot where touch device recognizes the touch. **d** The three-dimensional (3D) electromagnetic (EM) simulation of 100 MHz electromagnetic wave propagation in waveguide through PEDOT:PSS films of different concentrations. **e** Demonstrated writing application in which the thickness of a letter can be adjusted by varying the applied pressure. **f** Photograph of the setup of the magnetic resonance imaging (MRI)-compatible pressure sensor. An artificial beating orange is being scanned by MRI. **g** Photograph of a MHz-transparent pressure sensor attached to an artificial beating orange. **h** Setup for artificial beating orange. A syringe is used to control the size of the balloon inside the orange. **i** Changes in capacitance due to the artificial beats measured by the MRI-compatible pressure sensor during MRI scanning. **j** MR images of orange with a pressure sensor consisting of aluminum, pristine PEDOT:PSS, 15 wt.% PEDOT:PSS electrodes, and without a pressure sensor

We tested the transparent capacitive pressure sensor when attached directly to a touch-controlled display screen (Fig. 5a). The pressure sensor was connected to an Arduino board with a Bluetooth module (Supplementary Fig. 12). The Arduino system collected the capacitive responses by measuring the difference in the electric potential originating from the accumulated charge in the capacitive pressure sensor and transmitted the data to the mobile device via a Bluetooth module. Figure 5b is a schematic of the distribution of the electric field; the electric field between the driver and receiver passes through the capacitive pressure sensor attached to the mobile touchscreen without any electromagnetic interference, in the ‘untouched’ state (Fig. 5b, left-hand part), and the field is absorbed by a conducting object, i.e., a human finger, without any interference by the conducting electrodes of the pressure sensor, in the ‘touched’ state (Fig. 5b, right-hand part). The former exhibits no change in the magnitude of the electric field (Fig. 5b, bottom-left, ‘untouched’); the latter exhibits a significant change between driver and receiver (Fig. 5b, bottom-right, ‘touched’). When the conducting electrode blocks the electric field (Supplementary Fig. 13), the touchscreen overlaid with the pressure sensor cannot function properly; if external stimuli distort the electric field generated between the driver and the receiver electrodes, the touch accuracy will then be adversely affected. We can explain these different situations as follows: Fig. 5c shows the touch accuracy of the touchscreen with the pressure sensors consisting of different electrodes: pristine PEDOT:PSS electrodes (top) and 15 wt.% PEDOT:PSS electrodes (bottom, Fig. 5c). With the pristine PEDOT:PSS electrode, a decrease in the touch accuracy was observed; the red spot indicates the position at which the finger actually touches the screen, while the green spot indicates the position at which the touchscreen recognizes the touch. This inaccuracy is due to the high electrical conductivity of the electrode (744 S/m) that disturbs the electric field being used for touch recognition, as shown in Supplementary Fig. 13. In contrast, in the case of the 15 wt.% PEDOT:PSS electrode, the positions of the actual finger touch and touch recognition coincide (Supplementary Movie 1). This confirms that the electrical conductivity is a critical design parameter that increases the electric field transmission and accuracy of the touch recognition. We also explicitly modeled EM waves passing through PEDOT:PSS films of different concentrations using the 3D EM simulation program ANSYS HFSS (high-frequency structure simulator) under waveguide conditions (Fig. 5d); the color variation of the simulation indicates the intensity of the electric field, the dotted lines indicate the positions of the PEDOT:PSS electrodes, and the wave propagates from the ‘region I’ to the ‘region II’. Importantly, there is no noticeable change in the intensity of the maximum electric field passing through the 15 wt.% PEDOT:PSS, as opposed to the 50 wt.% (66% decrease) and pristine PEDOT:PSS (72% decrease), which is in reasonably good agreement with the observed experimental results. Note that there is an increase in the wave intensity in ‘region I’ in the case of the pristine PEDOT:PSS, compared to other concentrations, due to the wave reflection.

### Implementation for practical applications

Finally, we demonstrated the general applicability of pressure sensors integrated into a touchscreen. First, the pressure sensor was used as a 3D touch interface that serially writes a particular letter with thin and thick line widths. The thickness of the line is determined by the applied pressure at the beginning of a stroke; a constant capacitance ratio (Δ*C*/*C*_0_ = 0.7) was used as a threshold in the Arduino system to distinguish between soft and hard touches. When a soft touch was input through the pressure sensor, the line width was set to 0.03 cm, while when a hard touch was input, the line width was fixed to 0.2 cm until the end of the stroke. Indeed, as shown in Fig. 5e, the letters 'MML' with a variable line width were input to the display (Supplementary Movie 2): thin (Fig. 5e, left) and thick line width (Fig. 5e, right). Second, we demonstrated the applicability of our approach using a camera (Supplementary Fig. 14). When the capacitance change is larger than the threshold capacitance ratio (Δ*C*/*C*_0_ = 0.7), the magnification of the image increases to 120%, but decreases to 83% in the opposite case. Zoom-in and zoom-out were each performed twice; when the hard (or soft) touch is input, the magnification is increased (or reduced) to 144% (or 69%). In addition, as shown in Supplementary Movie 3, the images were successfully photographed and saved at two different scales.

We also demonstrated a MRI-compatible pressure sensor for biomedical applications (Fig. 5f). MRI images the internal structure of the human body by recognizing the magnetic field change corresponding to the radiofrequency range. When the body is placed in a strong magnetic field, such as a MRI scanner, the nuclear spin of hydrogen protons become aligned with the magnetic field in a Boltzmann distribution. This uniform alignment creates a magnetic vector oriented along the axis of the MRI scanner. When the radiofrequency source is switched off, the magnetic vector returns to its resting state, and this causes a radiofrequency (RF) signal which is used to create MR images^49^. Therefore, if the magnetic field and emitted RF signal for the scan are blocked by the conductors, an artifact may appear in the image. Using 15 wt.% PEDOT:PSS electrodes instead of conventional electrodes with high electrical conductivity can prevent this image distortion because it does not interfere with the RF signal used to implement the image. Due to the characteristics of RF wave-transparent electrodes, the pressure sensor does not degrade the quality of the MR image and can be operated simultaneously. For demonstration of a MRI-compatible pressure sensor, the pressure sensor with 15 wt.% PEDOT:PSS electrodes was attached to the surface of an orange, as shown in Fig. 5g. To deliver artificial beats from outside of the shielded room to the MRI subject (an orange), a balloon connected to a syringe was inserted into the orange (Fig. 5h and Supplementary Movie 4). When the syringe is pumped, the orange repeats expansion/contraction and beats regularly, as shown in Supplementary Movie 5. The pressure sensor connected to the LCR (inductance, capacitance, resistance) meter responds to volume changes in the orange, and as a result, the recognizable capacitance change was measured in real time with simultaneous MRI scanning (Fig. 5i and Supplementary Movie 6). When the MRI subject is attached with a pressure sensor using aluminum electrodes or pristine PEDOT:PSS electrodes, distortion of the MR image is observed (Fig. 5j, (i) and (ii)). Since the generated RF signal is reflected by the highly conductive electrode, an artifact occurs at the edge where the sensor is located. However, in the case of a pressure sensor using 15 wt.% PEDOT:PSS electrodes, such distortion does not occur, and the same quality image as when the sensor is not attached can be obtained (Fig. 5j, (iii) and (iv)). Since the sensor does not completely cover the surface of the orange, the farther it is away from the scanned center image will reduce the impact of artifacts, as shown in Supplementary Fig. 15. The realization of integrating pressure sensing with MRI not only demonstrates the practical applicability of medical sensing, but also indicates the possibility of interoperability between implantable wireless devices and body-external functional systems.

## Discussion

The above studies demonstrate that polymers that are transparent to low-frequency waves with a low plasma frequency could be used as conducting electrodes, especially in pressure sensors, and that these devices can be further integrated into touchscreen display devices and MRI scanning systems, providing a approach for addressing material design issues for device-to-device integration. In the preparation of the pressure sensor, we also adapted a nanoparticle-dispersed dielectric that optimizes the transparency (96.7%) and sensitivity (2.0 kPa^−1^, higher than that of the micropatterned counterpart^48,50,51^). The functionality and stability of the sensors with the low-frequency wave-transparent polymers was demonstrated using a pervasive OHP film as the substrate with no evident capacitance degradation over 10,000 load/unload cycles. We can argue that, in the context of 'mutually exclusive' materials properties^52–54^, this approach influences the direction of optically transparent and electrically conductive materials design. Given that the process for preparing a MHz frequency-transparent PEDOT:PSS involves solution-based polymer chemistry, there is the potential for aggressive device scaling without costly processing steps. By providing approaches to address the key issues in the context of: the relatively low conductivity that may cause certain issues (e.g., power dissipation and Joule heating) in applications other than a capacitor; the oxidation of PEDOT, when exposed to ambient air, where it can be reduced by encapsulation (e.g., glass plate or protective coating); and particle uniformity originating from the colloidal solution process, this lowers some of the barriers to transitioning the field of device-to-device compatibility from one of academic curiosity into one that can be focused on device design and production capabilities.

## Methods

### Fabrication of capacitive pressure sensor

An OHP film was treated with oxygen plasma at 100 W for 10 min to achieve strong adhesion between the film and a PEDOT:PSS layer. PEDOT:PSS (Clevios PH1000, Heraeus) was premixed with 85 wt.% ethanol and 1 wt.% surfactant (Triton X-100, Sigma-Aldrich). The PEDOT:PSS solution was spin-coated onto the film at 1000 rpm for 60 s and subsequently cured in a furnace at 70 °C for 6 h. For capacitance measurements, Ag-Ni woven conductive fabric (Solueta Co. Ltd., SILTEX, CNG type) was pasted onto the PEDOT:PSS electrodes to ensure a good electrical contact. Then, 500 nm silica beads (Cospheric LLC, SiO2MS-1.8 0.507 µm, CV < 6.4%, Sphericity > 99%) were suspended in ethanol at a ratio of 0.5 wt.%. Subsequently, the solution containing the silica beads was spin-coated onto the films at 3000 rpm for 30 s. PDMS (Sylgard 184, Dow Corning) and crosslinker were mixed (10:1 w/w), degassed, and subsequently diluted with heptane (CHROMASOLV Plus, for HPLC, 99%, Sigma-Aldrich) to prepare a 40 wt.% PDMS solution. The films were treated with oxygen plasma at 100 W for 10 min prior to the spin-coating of the PDMS solution. Subsequently, the PDMS solution was spin-coated onto the films at 1000 rpm for 30 s and then annealed at 70 °C for 24 h in a furnace. Finally, the upper OHP film/PEDOT:PSS electrodes were stacked onto the nanoparticle-dispersed half-cells facing each other, thus forming a capacitor.

### Characterization methods

The surface morphology and thickness of the PEDOT:PSS layer was analyzed using tapping-mode AFM (XE-150, Park Systems Corp.) with height and phase contrast. Single particles of the PEDOT:PSS film were examined by HAADF-STEM (JEM-F200, JEOL Ltd.). The particle size of the PEDOT:PSS in solution was examined by a particle size analyzer (ELS-1000ZS, Otsuka Electronics). Raman spectroscopy was done using a LabRAM Aramis unit (Horiba Jobin Yvon). XPS measurements were performed using a Thermo Scientific K-alpha spectrometer. The sheet resistance was measured using a Loresta GP MCP-T610 (Mitsubishi Chemical Analytech Co., Ltd.). *S*_21_ parameter measurements were done using an Anritsu E5071B vector network analyzer and calibrated with a E-calibration module over 50–200 MHz. The surface morphologies of the constituent layers were analyzed using a JSM-7001F field emission SEM (JEOL). The transmittance of the PEDOT:PSS films was analyzed using a V-650 ultraviolet visible scanning spectrophotometer (JASCO). A clean OHP substrate was used for a baseline scan to measure the transparency. The capacitance of the transparent capacitive pressure sensors was measured using a precision LCR meter (Agilent E4980A) under ambient conditions. The frequency was 1–100 kHz with an AC excitation voltage of 1 V. A universal manipulator (Teraleader) with a force resolution of 10 mN was set up with the LCR meter to undertake pressure-dependent capacitance change measurements. MRI was performed using a 3.0 T scanner (Ingenia CX, Philips Medical Systems, Best, The Netherlands) with a 32-channel sensitivity-encoding head coil. The parameters for the T_1_ fast field echo (FFE) sequence were as follows: repetition time (TR), 5 ms; echo time (TE), 275 ms; flip angle, 80 degrees; matrix size, 512 × 512; pixel resolution, 0.20 × 0.20 mm^2^; slice thickness, 3 mm; and acquisition time, 2 min 6 s.

## Supplementary Information

Supplementary Information

Description of Additional Supplementary Files

Supplementary Movie 1

Supplementary Movie 2

Supplementary Movie 3

Supplementary Movie 4

Supplementary Movie 5

Supplementary Movie 6

## Data Availability

The data that support the findings of this study are available from the corresponding author upon request.
