# Supplementary material for: Megahertz-wave-transmitting conducting polymer electrode for device-to-device integration
Source: Nat Commun. 2019 Feb 8;10:653. doi: 10.1038/s41467-019-08552-z (PMC6368642; doi:10.1038/s41467-019-08552-z)
Supplement: Supplementary file 1 — Supplementary Information [file 41467_2019_8552_MOESM1_ESM.pdf]

# Supplementary Information

## Megahertz-Wave-transmitting Conducting Polymer Electrode

## for Device-to-Device Integration

Kim, T. *et al.*

**This PDF file includes:**

Supplementary Figures 1−19

Supplementary Notes 1−2

**Supplementary Figures**

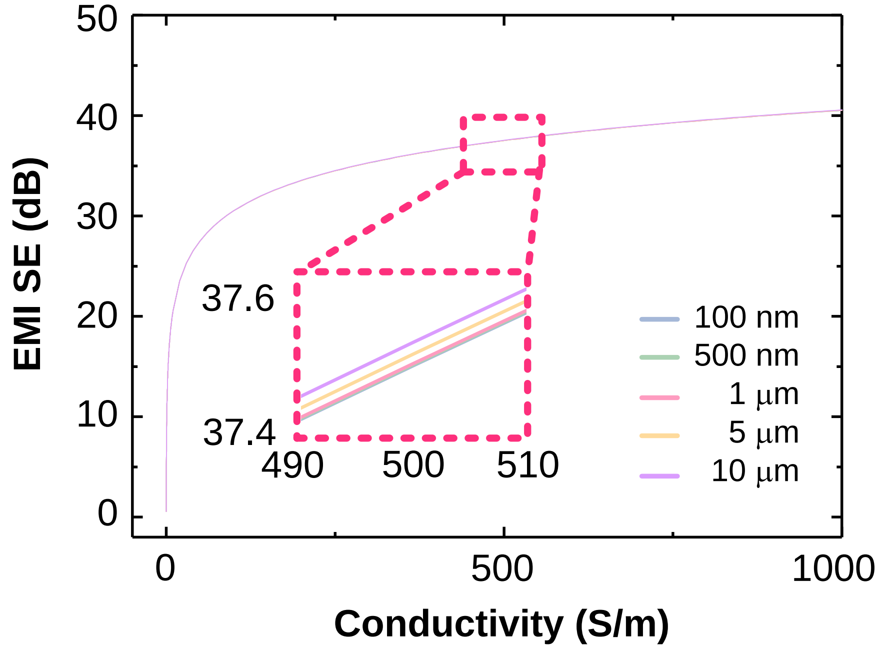


**Supplementary Figure 1. EMI shielding efficiency (SE) of conductors.** When the EMI SE is calculated for conductors with a thickness in the order of nanometers to micrometers with 100 MHz electromagnetic waves, the thickness dependence of the EMI SE is extremely small (inset) and, therefore, the conductivity is the dominant factor determining the EMI SE.

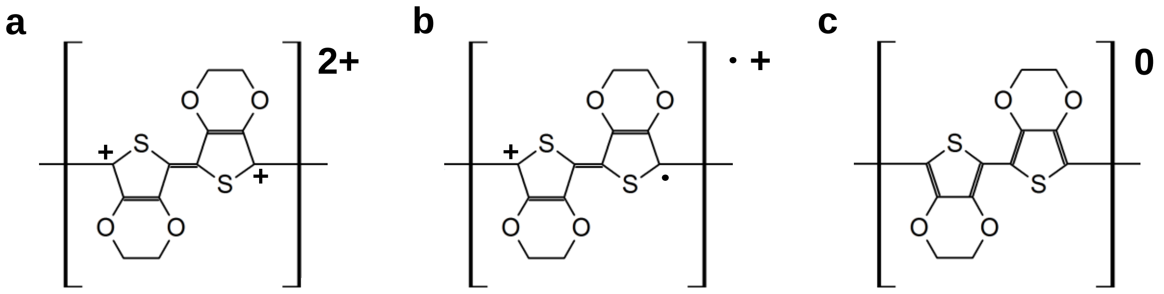


**Supplementary Figure 2. Schematic of transition state of PEDOT chains.** PEDOT is transformed from a quinoid structure to a benzoid structure as the pH increases. The replacement of $H^+$ causes a shift in the equilibrium from the (**a**) bipolarons, (**b**) polarons states to a (**c**) neutral state.

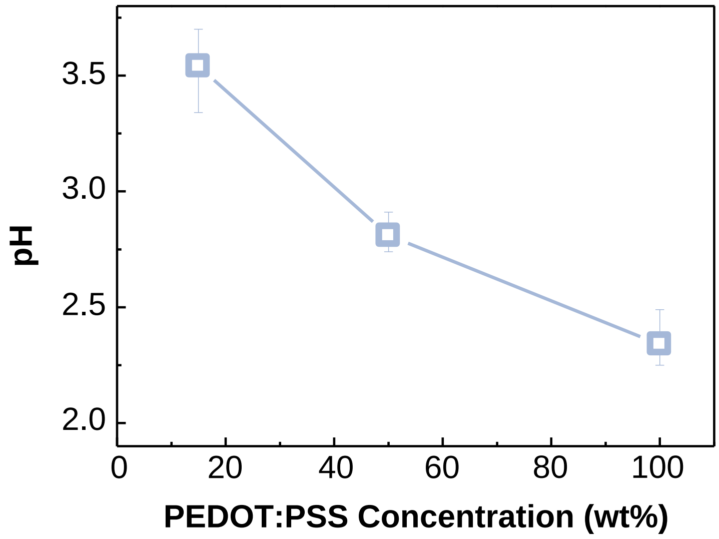


**Supplementary Figure 3. pH of PEDOT:PSS/ethanol solution.** Pristine PEDOT:PSS has a strong acidity of about pH 2.34. As ethanol is added, the ion concentration decreases due to an increase in the amount of the solvent, after which the pH gradually increases. The error bar represents data range.

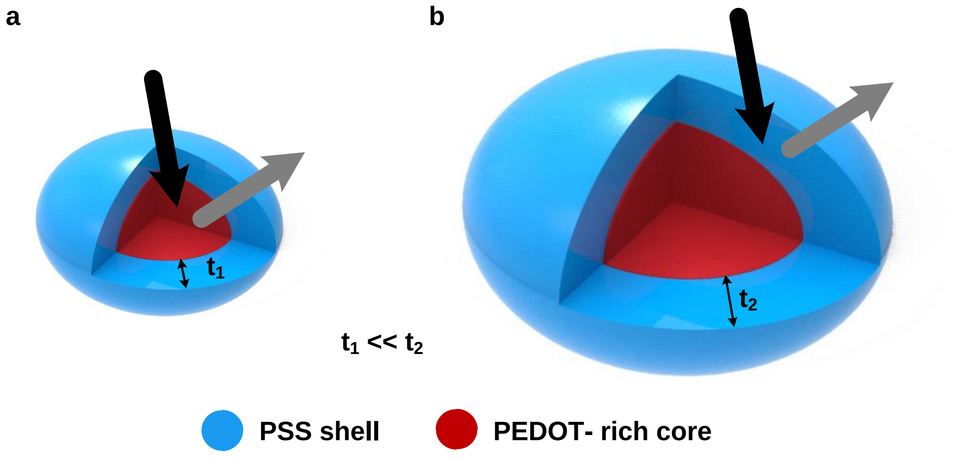


**Supplementary Figure 4. Illustration of difference in XPS analysis regions.** The reachability of the PEDOT-rich core for the photons is determined by the thickness of the PSS shell. (**a**) Schematic of photon reaching a PEDOT-rich core through a PSS shell. (**b**) Schematic of a PSS shell being thick enough to prevent photons from reaching the PEDOT-rich core. In this case, the XPS spectra reveal the composition of the PSS shell.

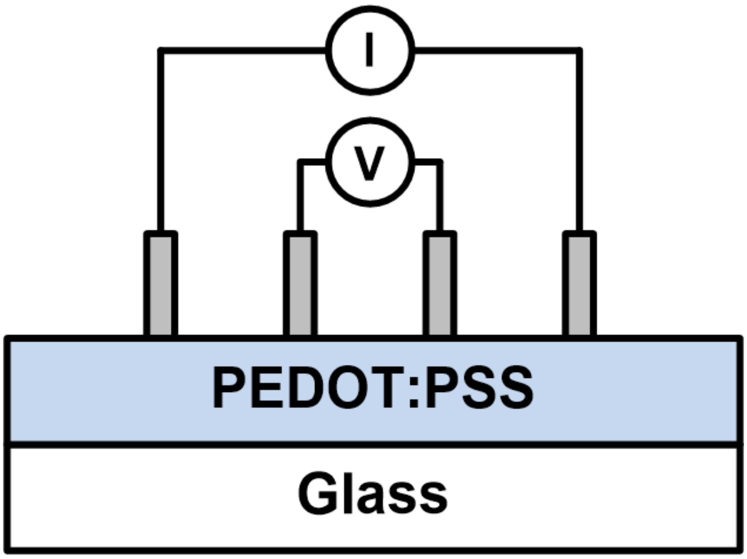


**Supplementary Figure 5. Illustration of 4-point probe measurement.** Measure the sheet resistance of the spin-coated PEDOT:PSS films using 4-point probe measurement. The resistivity and electrical conductivity calculated by using the measured sheet resistance and the thickness of the film.

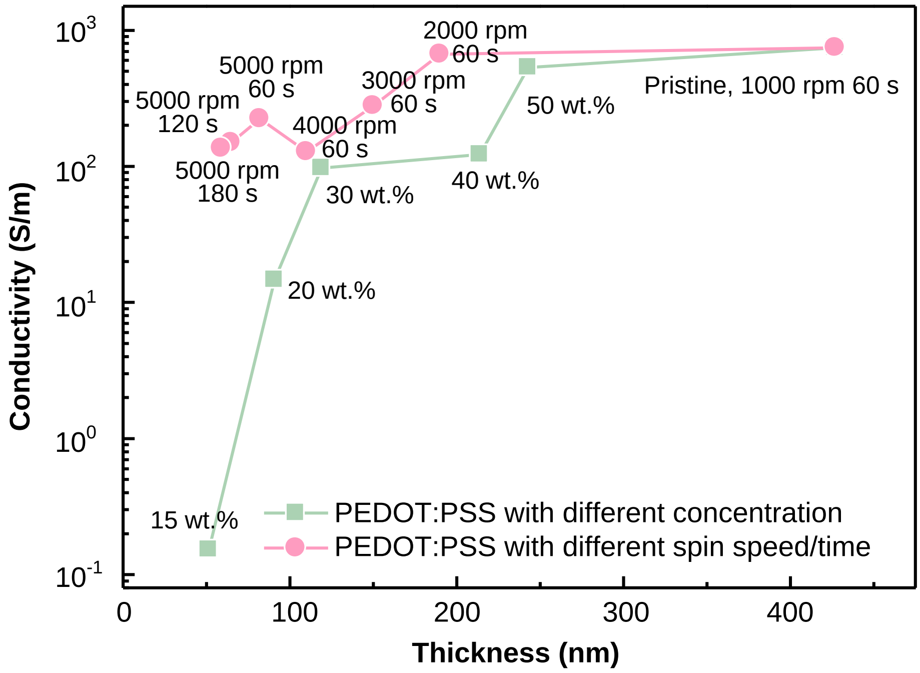


**Supplementary Figure 6. Difference in degree of electrical conductivity decrease.** The red line corresponds to pristine PEDOT:PSS films with reduced thickness by increasing the spin speed and time ('1000 rpm 60 s'–'5000 rpm 180 s'). The green line corresponds to PEDOT:PSS films with reduced thickness by decreasing the concentration of the spin-coating PEDOT:PSS solution (100 wt.% (pristine) to 15 wt.%). Both methods reduced the thickness to about 50 nm. The decrease in the conductivity was very rapid when the concentration was adjusted (decreasing by almost four orders of magnitude). In contrast, the conductivity decreases by less than one order of magnitude as a result of controlling the spin speed/time to decrease the thickness.

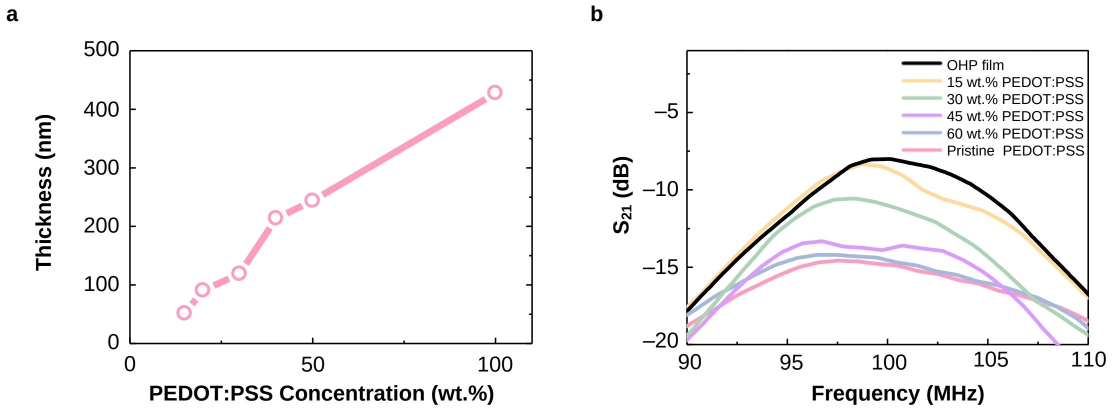


**Supplementary Figure 7. Thickness and MHz transmittance of PEDOT:PSS films.** (**a**) Thickness of PEDOT:PSS film formed by spin-coating at 1000 rpm for 60 s with different concentrations. The PEDOT:PSS was diluted from a pristine solution to 15 wt.% and the thickness gradually decreased from 430 to 50 nm. (**b**) Measurement of $S_{21}$ parameters of PEDOT:PSS films prepared at different concentrations. The variability in the $S_{21}$ in this case was about 6.2 dB at 100 MHz.

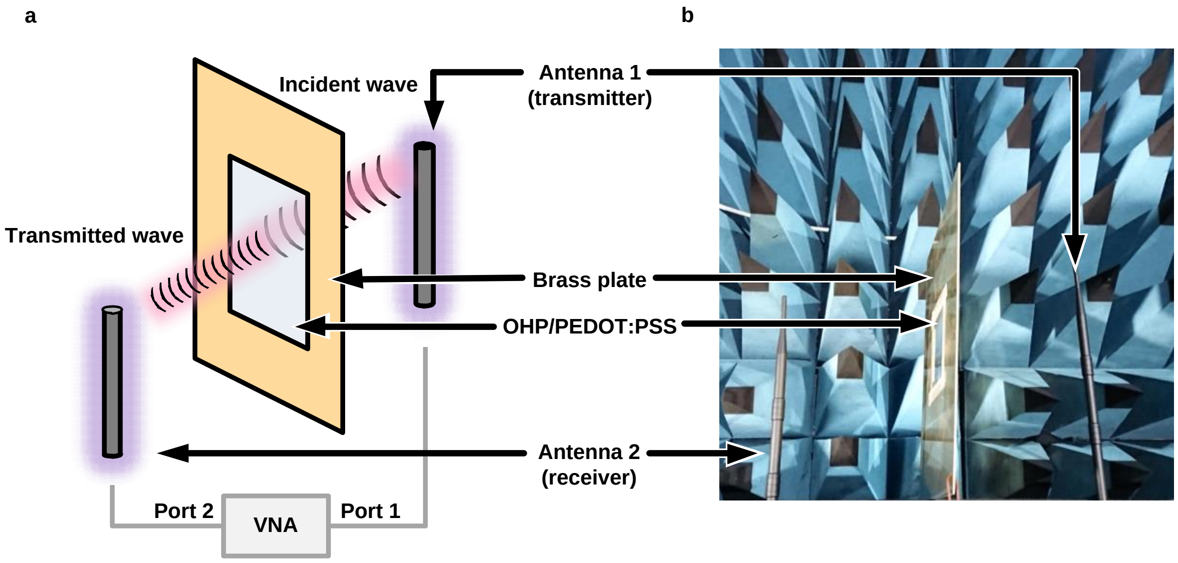


**Supplementary Figure 8. Schematic and photograph of measurement setup for $S_{21}$. (a)** Schematic of measurement of $S_{21}$ parameter. The antennae are placed 10 cm from the brass plate to which the OHP/PEDOT:PSS film is attached, and both antennae are connected to the vector network analyzer. (**b**) $S_{21}$ parameter measurement setup. Experiments were conducted in an anechoic chamber to suppress the effects of unintentional reflection.

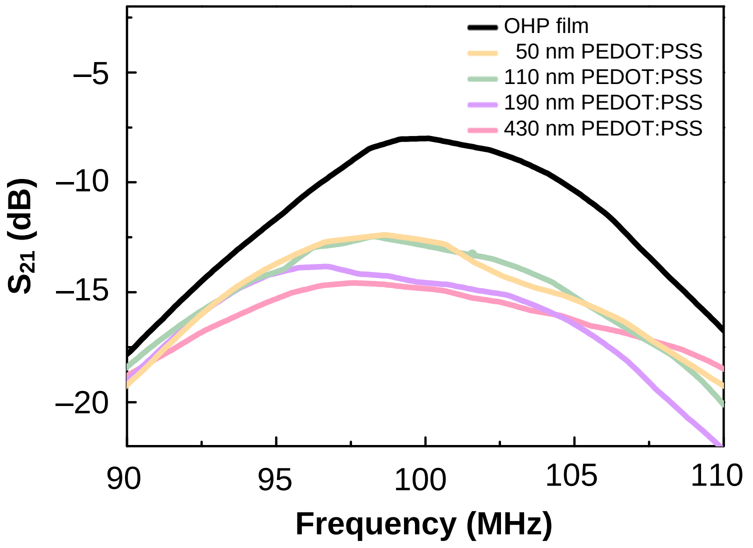


**Supplementary Figure 9. MHz transmittance of pristine PEDOT:PSS films.** Measurement of $S_{21}$ parameters of PEDOT:PSS films of different thicknesses. All the films were formed by spin-coating pristine PEDOT:PSS. The decrease in the thickness was attained by increasing the spin speed and time. The variability in the $S_{21}$ in this case was about 2.3 dB at 100 MHz. This shows less variability than when the thickness is lowered to the same level (50 nm) through the decreasing PEDOT:PSS concentration.

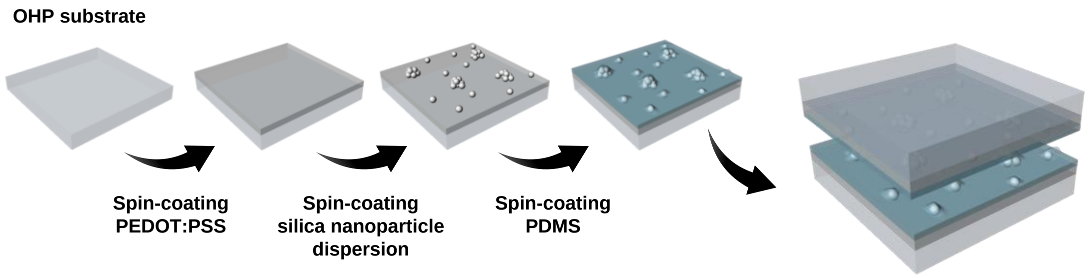


**Supplementary Figure 10. Fabrication of capacitive pressure sensor.** The sensor is based on an OHP film and the fabrication consists only of spin-coating processes. First, the substrate is coated with a PEDOT:PSS solution which serves as an electrode. Then, silica nanoparticles are formed to increase the air gap between both electrodes and increase the change in the effective permittivity. In the final step, a PDMS layer was formed as a dielectric layer. These two layers are attached facing each other to form a capacitive pressure sensor.

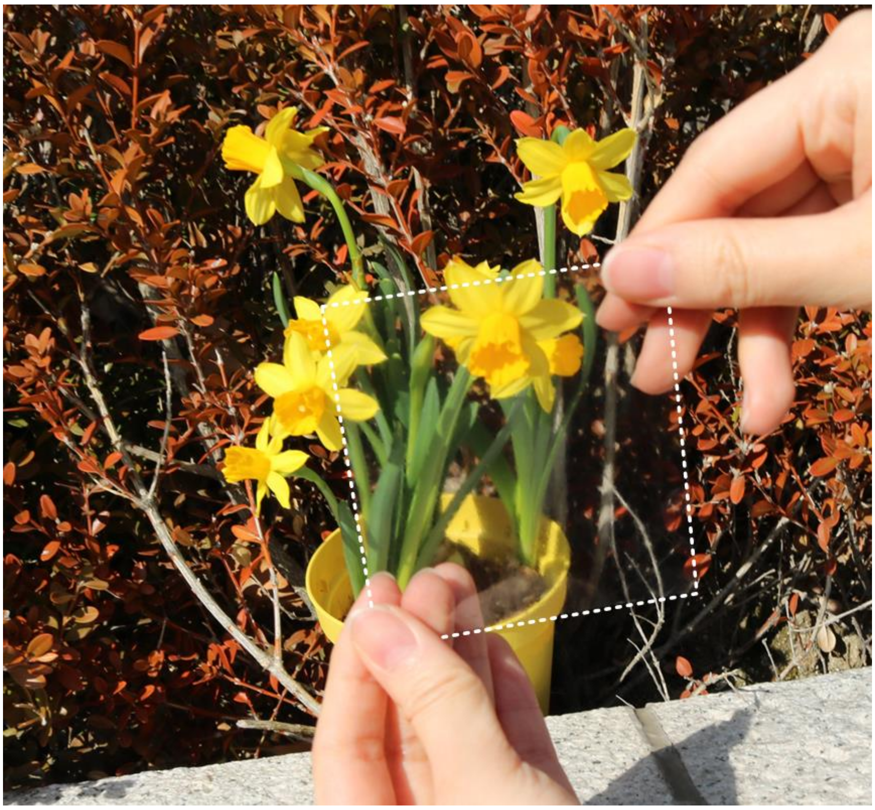

**Supplementary Figure 11. Large-area sensor layer formed on the OHP film substrate.** The sensor has very high transparency (> 96.7%) and maintains this level of transparency even when fabricated at very large scales. The capacitive pressure sensor is indicated by the white dotted line.

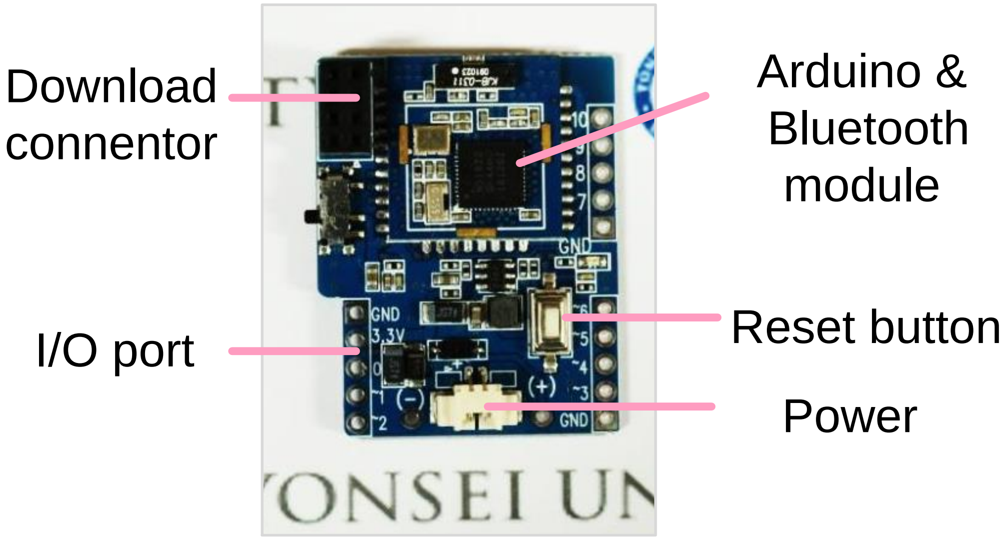


**Supplementary Figure 12. Arduino board with Bluetooth module.** The Arduino board is used without a continuous USB connection once the code has been written using the download connector. A button cell is connected to the power terminal, allowing the module to be operated without a wired power supply. The sensor is connected to the input/output (I/O) port by soldering.

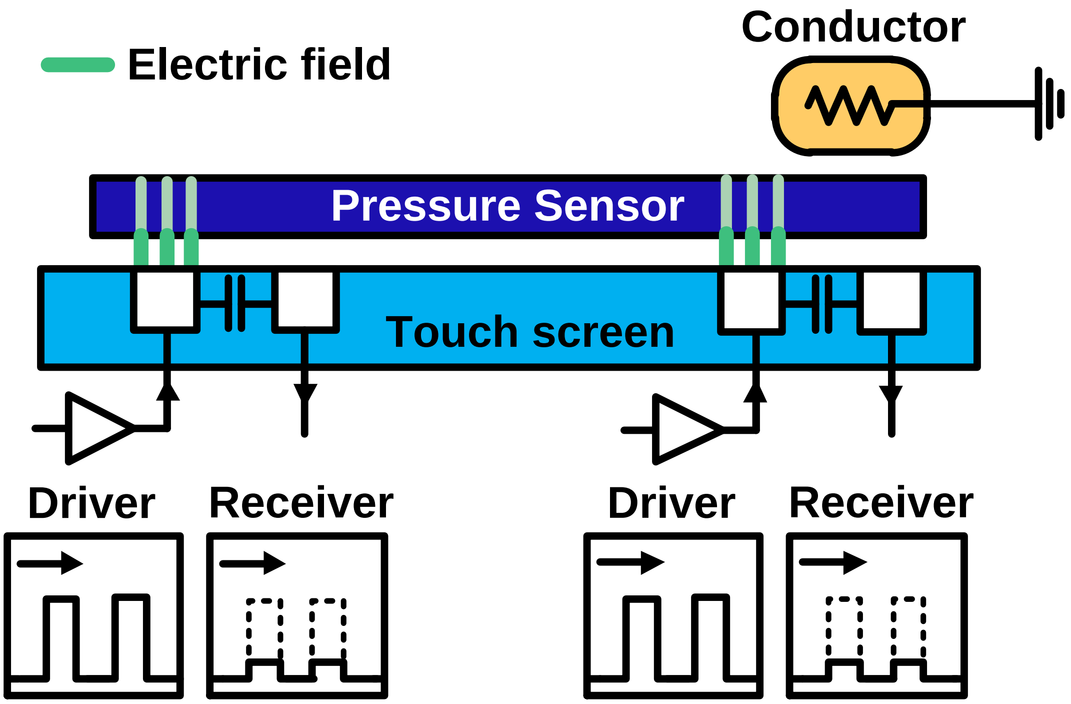


**Supplementary Figure 13. Electric field distribution of sensor using normal electrode.** Unlike that shown in Fig. 5a, the electric field generated from the touch screen is completely blocked by the electrodes constituting the sensor. Therefore, any reduction in the electric field is recognized by all the receivers, and the location of any touch cannot be distinguished.

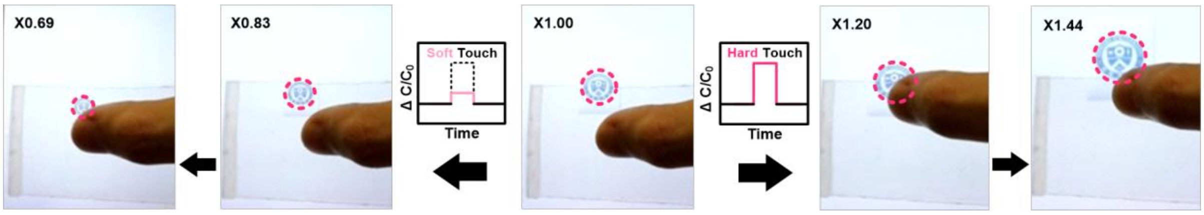


**Supplementary Figure 14. Demonstration of camera applicability.** Mobile phone application in which the degree of zoom-in/out is determined by the applied pressure; zoom-in (120%) and zoom-out (83%) is up to 2 times respectively.

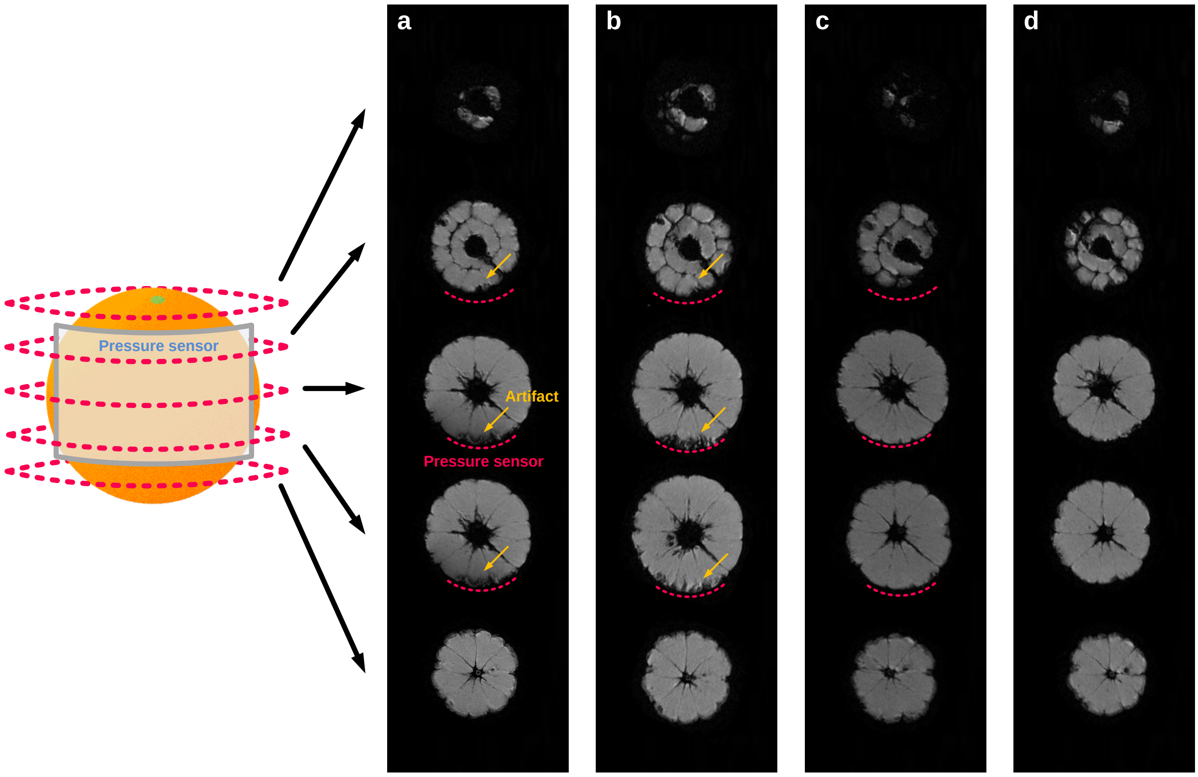


**Supplementary Figure 15. MRI data sets of oranges with pressure sensors.** The black artifact is observed at the bottom of the image (sensor attached direction) when pressure sensors containing (**a**) aluminum and (**b**) pristine PEDOT:PSS electrodes are attached. However, no artifacts are found when a pressure sensor containing (**c**) 15 wt.% electrodes is attached and when (**d**) no sensor is attached.

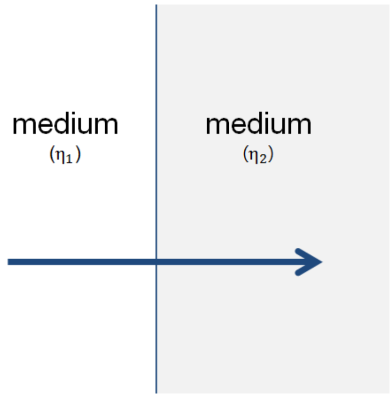


**Supplementary Figure 16. Electromagnetic wave passing through the boundary between two media.** When the electromagnetic wave passes through the interface, the intrinsic impedance changes.

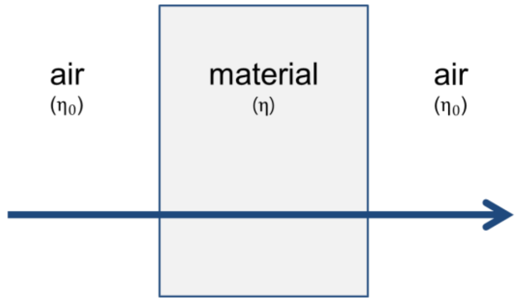


**Supplementary Figure 17. Electromagnetic wave passing through the material.** When electromagnetic waves pass through the material, the intrinsic impedance changes twice.

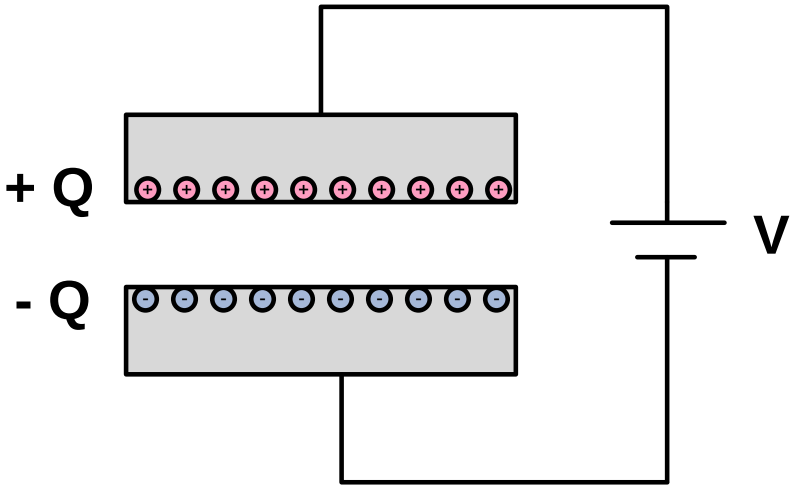


**Supplementary Figure 18. Schematic of capacitor charged with voltage V.** When voltage is applied across the capacitor, charge accumulates on the electrode surface.

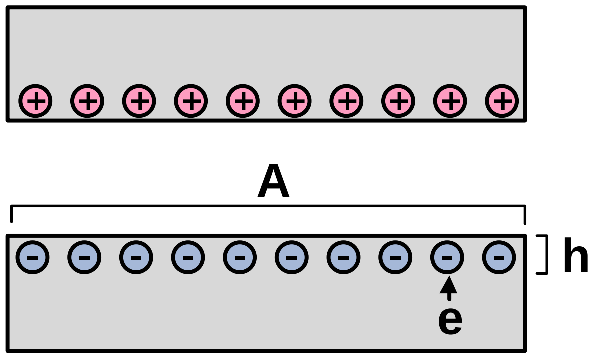


**Supplementary Figure 19. Depth contributing to charge in a charged capacitor.** The accumulated charge is very thinly placed on each plate surface.

## Supplementary Notes

### Supplementary Note 1: Determining the transmission and reflection ratio.

When an electromagnetic wave travels through a medium, the electric and magnetic fields are perpendicular to each other and also perpendicular to the direction of propagation. The intrinsic impedance is defined as the ratio of the electric field to the magnetic field in a far field. The intrinsic impedance of a material $\eta$ is expressed by:

$$\eta = \sqrt{\frac{i\omega\mu}{\sigma + i\omega\varepsilon}} \tag{1}$$

($\omega$: angular frequency of wave, $\mu$ : permeability of material, $\varepsilon$: permittivity of material, $\sigma$: conductivity of material)

Since the material is a good conductor, it is reasonable to make the following approximation:

$$\eta = \sqrt{\frac{i\omega\mu}{\sigma + i\omega\varepsilon}} = \sqrt{\frac{i\omega\mu}{\sigma}}\sqrt{\frac{1}{1 + i\omega\varepsilon/\sigma}}$$

$$\approx \sqrt{\frac{i\omega\mu}{\sigma}} \tag{2}$$

Furthermore, the intrinsic impedance of free space (air) $\eta_0$ is

$$\eta_0 = \sqrt{\frac{i\omega\mu}{\sigma + i\omega\varepsilon}} = \sqrt{\frac{i\omega\mu_0}{0 + i\omega\varepsilon_0}} = \sqrt{\frac{\mu_0}{\varepsilon_0}} \approx 377\ \Omega \tag{3}$$

($\varepsilon_0$: vacuum permittivity, $\mu_0$: vacuum permeability)

1) Wave passing from medium ($\eta_1$) to other medium ($\eta_2$)

When an electromagnetic wave passes through the interface between two media with different intrinsic impedances (Supplementary Fig. 15), a portion of it will be reflected while the remainder will be transmitted. The intensity of the reflected part relative to the incident wave is represented by the reflection coefficient Γ.

If the wave passes from one medium ($\eta_1$) to another ($\eta_2$), the reflection coefficient ($\Gamma_{12}$) at the interface of any medium (material) is given by:

$$\Gamma_{12} = \frac{\eta_1 - \eta_2}{\eta_1 + \eta_2} \tag{4}$$

Then, the intensity of the electromagnetic wave transmitted through the interface is

$$1 - \Gamma_{12} = \frac{2\eta_2}{\eta_1 + \eta_2} \tag{5}$$

2) Wave passing through a free-standing film

Assuming that the film is freestanding in the air, the electromagnetic wave propagates through the interfaces between the air/film and film/air (Supplementary Fig. 16). At each interface, the intensity decreases at each interface by a ratio of $1 - \Gamma_{air/film}$ and $1 - \Gamma_{film/air}$, resulting in the following ratio of transmitted wave intensity, $E_t$, compared to the incident wave intensity, $E_i$.

$$\left|\frac{E_t}{E_i}\right| = \left|\frac{E_t}{E_1}\frac{E_1}{E_i}\right| = \left|(1-\Gamma_{\text{air/film}})(1-\Gamma_{\text{film/air}})\right| = \left|\frac{2\eta}{\eta_0+\eta} \bullet \frac{2\eta_0}{\eta_0+\eta}\right| = \left|\frac{4\eta\eta_0}{(\eta_0+\eta)^2}\right| \quad (6)$$

($E_1$: intensity of the wave propagating inside material)

Since the material from which the film is formed is a good conductor, $\eta_0 \gg \eta$, the ratio of the transmitted wave intensity to incident wave intensity can be approximated as:

$$\left|\frac{E_t}{E_i}\right| = \left|\frac{4\eta\eta_0}{(\eta_0+\eta)^2}\right| \approx \left|\frac{4\eta\eta_0}{{\eta_0}^2}\right| = \left|\frac{4\eta}{\eta_0}\right| \quad (7)$$

Therefore, using Eqs. (2) and (3), the loss ratio due to reflection, $R$, is obtained as:

$$R = 1 - \left|\frac{E_t}{E_i}\right| = 1 - \left|\frac{4\eta}{\eta_0}\right| = 1 - \frac{4}{377}\sqrt{\frac{\omega\mu}{\sigma}} \quad (8)$$

Furthermore, the intercept frequency of the reflection ratio ($R$ = 0), $f_{R=0}$, can be calculated as:

$$f_{R=0} = \left(\frac{377}{4}\right)^2 \frac{\sigma}{2\pi\mu} \quad (9)$$

The electrodes inside the actual capacitive pressure sensor do not exist freestanding, but are placed between the insulators. Since the electrical conductivity of insulator layers (e.g., PDMS and OHP film) is negligibly low, the insulator has intrinsic impedance similar to that of air (377 Ω). Therefore, the wave intensity loss due to reflections at the interface between insulator layers and air is negligible ($\Gamma_{12} = \frac{\eta_1-\eta_2}{\eta_1+\eta_2} \approx 0$). For the same reason, the loss due to reflections at the insulator and conductor interfaces is approximated to the interface between air and the conductor.

**Supplementary Note 2: Charge accumulation depth of capacitor.**

When a capacitor is connected to a circuit, an electrical potential difference $V$ is generated between both plates constituting the capacitor with capacitance $C$. At this time, charges accumulate on the surface of each plate to maintain this potential difference (Supplementary Fig. 17). The charge stored on both plates of the capacitor, $Q$, is as follows:

$$Q = CV \tag{10}$$

In this case, the number of electrons that rise to the surface, $N$, is determined by the charge stored on either plate, $Q$, and the electron charge $e$.

$$N = Q/e = CV/e \tag{11}$$

At this time, the electrons are supplied by the atoms of the plate. Thus, the volume of the conductor that should provide electrons, $U$, is calculated as follows.

$$U = N/n = CV/en \tag{12}$$

($n$: density of electrons in the conductor)

Therefore, the depth of the plate contributing to the charge, $h$, is calculated by dividing by the area of the plate $A$ (Supplementary Fig. 18).

$$h = U/A = CV/enA \tag{13}$$

Applying the conditions used to measure the sensor's capabilities,

($C$ = 30pF, $A$ = $10^{-4}$ $m^2$, $V$ = 5 V, $e$ = 1.60217662 × $10^{-19}$ C, $n_{PEDOT:PSS}$ = 2.25 × $10^{20}$ /$cm^3$):

$$h = \frac{CV}{enA} = \frac{(30 \times 10^{-12}\text{F}) \cdot 5\text{V}}{(1.60217662 \times 10^{-19}\text{ C}) \cdot (2.25 \times 10^{26}\, m^{-3}) \cdot 10^{-4}\text{ m}^2}$$

$$= 4.161 \times 10^{-14}\, m = 0.04161\text{ pm} \qquad (14)$$

In other words, the depth of a conductor in which electrons can move to the surface is of a very small range of < 1 pm, such that even if the thickness of the capacitor is very thin, it does not affect the sensing performance.
