# Supplementary material for: Megahertz-wave-transmitting conducting polymer electrode for device-to-device integration
Source: Nat Commun. 2019 Feb 8;10:653. doi: 10.1038/s41467-019-08552-z (PMC6368642; doi:10.1038/s41467-019-08552-z)
Supplement: Supplementary file 2 — Description of Additional Supplementary Files [file 41467_2019_8552_MOESM2_ESM.pdf]

Description of Additional Supplementary Information

File Name: Supplementary Movie 1.

Description: Differences in touch accuracy depending on electrode of the attached pressure sensor. The pressure sensors on the touchscreen are coated with different electrodes: pristine PEDOT:PSS and 15 wt.% PEDOT:PSS. Upon touching the area on which the pristine PEDOT:PSS film is applied, the device does not recognize where it is actually touched regardless of whether the touch is long or short, with the point at which the touchscreen recognizes the touch (green dot) moving unstably. Conversely, when a pressure sensor coated with 15 wt.% PEDOT:PSS is attached, the touch accuracy is the same as the region without any film.

File Name: Supplementary Movie 2.

Description: Writing application in which the thickness of a letter can be changed by varying the applied pressure. With the application of pressure to a film attached to a touch screen, the position of the touch is recognized on the touch screen. By integrating these, a thin line is drawn when writing with low pressure. When writing the same text ("MML") with high pressure, it is expressed in bold letters.

File Name: Supplementary Movie 3.

Description: Camera application that zooms in/out according to the applied pressure. When a touch is recognized on the screen, the attached film recognizes the level of the pressure. When a low pressure is recognized, the camera zooms out. When a high pressure is applied, the camera zooms in. Images were acquired by zooming in (out) by ×1.44 (×0.69) compared with the initial state.

File Name: Supplementary Movie 4.

Description: Artificial beating using a balloon for demonstration of a MRIcompatible pressure sensor. When the syringe is pumped in a closed system consisting of a syringe, tubing, and balloon, the internal pressure changes. Therefore, the volume of the balloon is determined by the pressure applied to the syringe. Repetitive syringe pumping makes the balloon expand and contract, resulting in artificial beating.

File Name: Supplementary Movie 5.

Description: Artificial beating of orange for demonstration of a MRIcompatible pressure sensor. When the syringe is pumped after inserting the balloon inside the orange, an artificial beating at the same rate as the syringe is observed on the surface of the orange.

File Name: Supplementary Movie 6.

Description: Demonstration of MRI-compatible pressure sensor with simultaneous MRI scanning. MRI scans of the artificial beating orange with sensor attached. Artificial beats begin simultaneously with the MRI scan and the attached sensor outputs capacitance in real time. The amount of capacitance changes due to the volume change of the orange.
